# Prolonged follow‐up on lenalidomide‐based treatment for mucosa‐associated lymphoid tissue lymphoma (MALT lymphoma)—Real‐world data from the Medical University of Vienna

**DOI:** 10.1002/hon.2647

**Published:** 2019-07-28

**Authors:** Barbara Kiesewetter, Wolfgang Lamm, Ortrun Neuper, Marius E. Mayerhoefer, Ingrid Simonitsch‐Klupp, Markus Raderer

**Affiliations:** ^1^ Department of Medicine I, Clinical Division of Oncology Medical University of Vienna Vienna Austria; ^2^ Department of Biomedical Imaging and Image‐guided Therapy, Division of Nuclear Medicine Medical University of Vienna Vienna Austria; ^3^ Department of Pathology Medical University of Vienna Vienna Austria

**Keywords:** extranodal lymphoma, IMiD, immunomodulatory treatment, lenalidomide, MALT lymphoma

## Abstract

Based on results of two pilot trials, lenalidomide (LEN) was found to be active and safe as monotherapy and showed an increased response rate of 80% in combination with rituximab (R) for patients with mucosa‐associated lymphoid tissue (MALT) lymphoma. While initial results were promising, there are currently no data on long‐term outcome, and larger international phase II/III trials on LEN for indolent lymphoma lack specific subgroup analyses. Thus, we have systematically analyzed 50 patients treated with LEN‐based therapy (LEN‐monotherapy n = 16, R‐LEN n = 34) at the Medical University of Vienna 2009 to 2019 and investigated long‐term outcome and relapse patterns. At a follow‐up of more than 5 years (median 68 months), 54% of patients are free of relapse, and estimated median progression‐free survival (PFS) was 72 months (95%CI 49‐96). There was no difference in PFS according to stage of disease, i.e. localized versus disseminated disease (*P* = .67) and previous systemic treatment (*P* = .16). Interestingly, but with the caveat of the limited number of patients included in this series, primary extragastric disease had a superior PFS compared with gastric lymphoma (*P* = .04) and also depth of response, i.e. complete or partial response versus stable disease was associated with significantly prolonged PFS (*P* = .01). We documented four patients (8%) with pronounced improvement of response during follow‐up including three patients initially rated as partial remission and finally achieving complete remission at 12 to 32 months. This highlights the potential of delayed responses to LEN treatment. Estimated overall survival at 5 years was excellent at 92%. These “real‐world” data confirm long‐term activity of LEN in MALT lymphoma.

## 1 INTRODUCTION

Mucosa‐associated lymphoid tissue lymphoma (MALT lymphoma) constitutes a distinct type of indolent lymphoma, histologically characterized by a marginal zone B‐cell phenotype of CD20+ CD5− CD10− cyclinD1−, +/− light chain restriction, and variable presence of plasmacytic differentiation.^1^ MALT lymphoma cells develop following chronic antigenic stimulation highlighted by the association with chronic infections such as Helicobacter pylori (HP) for gastric MALT lymphoma and autoimmune disorders, e.g. autoimmune thyroiditis (Hashimoto's disease) and Sjogren's syndrome for various localizations.^2^, ^3^, ^4^, ^5^ According to the common conception, both conditions result in a multistep process of accrual of (HP‐antigen) specific T‐cells and production of proinflammatory immune mediators triggering a clonal B‐cell proliferation via nuclear transcription factor kappa B (NF‐ΚB) pathway activation. This concept has been further underlined by successfully using HP‐eradication for therapy of gastric MALT lymphoma, leading to 80% long‐term remissions in gastric lymphoma following elimination of the bacteria, thus proving a striking dependency of MALT lymphoma cells on the tumor microenvironment.^6^, ^7^

Following these data, HP‐eradication is the treatment standard for localized gastric MALT lymphoma, but there is still no clear explicit therapeutic algorithm for extragastric, disseminated, and HP‐refractory disease.^8^, ^9^, ^10^ Radiotherapy is widely used and may result in potential cure (or at least long‐term remission) for patients with localized disease,^11^, ^12^ but also chemotherapy‐based regimens such as chlorambucil or bendamustine +/− rituximab (R) are highly effective irrespective of stage.^13^, ^14^ Based on the pathogenesis, however, investigation of immunomodulatory therapies appears feasible in patients with MALT lymphoma.

As of 2019, a variety of chemotherapy‐free and/or immunomodulatory treatment concepts have been evaluated for MALT lymphoma including anti‐CD20‐antibody monotherapy with rituximab (R) or ofatumumab, the proteasome inhibitor bortezomib, tyrosine kinase inhibitor ibrutinib, and the macrolide‐antibiotics clarithromycin and azithromycin.^15^, ^16^, ^17^, ^18^, ^19^ However, results reported so far were mostly phase II data with promising results in terms of response rates and toxicity profiles, but there is a lack of prolonged follow‐up reports and the durability of responses beyond the defined treatment protocol. In addition, some agents were tested in and published from mixed collectives of marginal zone lymphoma (MZL) patients also including splenic and nodal MZL patients, what may further bias interpretation of results due to post‐hoc analyses.

Starting in 2005 and acknowledging the importance of immunomodulatory drug development in this specific disease, our department has extensively evaluated the effect of IMiDs in MALT lymphoma, as these compounds were reported to be highly effective in multiple myeloma, a disease not dissimilar to MALT lymphoma considering the close relationship of both malignancies.^20^, ^21^ Whereas an initial pilot on eight patients treated with thalidomide failed and resulted in no response during the observation period,^22^ prolonged follow‐up outside the protocol showed delayed effects of therapy in some patients. As opposed to this, treatment with the second generation drug lenalidomide (LEN) turned out as more promising with documented overall response rates (ORR) >60% for LEN‐monotherapy and improved outcome of 80% ORR for combination treatment with R‐LEN in the AGMT‐MALT2 study, an Austrian multicenter trial (n = 46 patients).^23^, ^24^ Toxicity profiles were favorable for both treatment schedules with moderate skin adverse events being the main reported side effect. In addition to our data, there is also evidence from larger phase II and III studies including mixed collectives of indolent lymphoma that LEN is an active and safe drug in this disease, but results are once again inconsistent due to only small subgroups of MZL included, particularly in the relapsed/refractory setting.^25^, ^26^, ^27^

To date, we have treated a total of 50 MALT lymphoma patients with LEN‐based treatment at the Medical University of Vienna, a tertiary referral center for patients with extranodal lymphoma of MALT type. Considering above discussed caveats in the current trial landscape of MALT lymphoma, the objective of this article is to present here consistent “real‐world” follow‐up data on response, relapse patterns, and safety with a median follow‐up of more than 5 years.

## 2 METHODS

For the current investigation, we have retrospectively analyzed patients treated with LEN‐based therapy for histologically verified MALT lymphoma at the Medical University of Vienna, Clinical Division of Oncology, between 2009 and 2019. All diagnoses were made by a reference hematopathologist according to the definition of MALT lymphoma as outlined in the WHO classification for tumors of hematopoietic and lymphoid tissues.^1^ As LEN is currently not approved for the treatment of extranodal MZL in Austria, all patients were treated within phase II trials approved by the ethical committee of the Medical University of Vienna and so was the current retrospective analysis. Initial outcome of treatment/studies has been published as referenced,^23^, ^24^ but the current investigation assessed only those patients treated and followed at our center, excluding patients from the multicenter AGMT‐MALT2 trial managed at other institutions. Current (follow‐up) data have been extracted from electronical and/or paper‐based medical records routinely captured and stored at the Medical University of Vienna.

### 2.1 LEN‐based treatment

Treatment regimens applied included LEN‐monotherapy, i.e. 25 mg daily from day 1 to 21 in a 28‐day cycle (maximum of six cycles) or R‐LEN consisting of R at 375 mg/m^2^ day 1 plus LEN 20 mg daily day 1 to 21 in a 28‐day cycle (maximum of eight cycles). Response was evaluated after cycles 3, 6, and 8 if applicable, and outcome was classified for best response based on either radiological or GELA histological criteria^28^ depending on primary localization, and documented as complete remission (CR), partial remission (PR), stable disease (SD), or progressive disease (PD). For more detailed information on inclusion/exclusion criteria, response classification, and dose reduction schedules, see the initially published trials.^23^, ^24^

### 2.2 Evaluated data

Data of interest for this analysis included basic patient characteristics, i.e. sex, age at treatment start, primary localization of MALT lymphoma, stage of disease, presence of risk factors/disease relevant comorbidities including lactate dehydrogenase (LDH) levels, beta‐2 microglobulin (B2M), presence of an autoimmune disorder and HP status, and histological features, i.e. documentation of plasmacytic differentiation. Furthermore, MALT‐IPI scores as recently published were retrospectively assessed.^29^ In terms of treatment‐related data, we documented initial outcome as stated above, potential late remissions if best response had changed during prolonged follow‐up, occurrence of relapses and respective site, further treatment, progression‐free survival (PFS) estimated from LEN treatment start to date of progression, time to relapse (TTR) from treatment start, time to next treatment (TTT) defined from end of LEN to start of next therapy, and finally duration of follow‐up, overall survival (OS) and current status of disease (alive with/without lymphoma, dead) including cause of death and transformation rate if applicable.

### 2.3 Statistical analysis

Statistical analysis was performed using IBM statistics for Mac OS version 25.0 (IBM, Armonk, NY). Metric data were described using median and range or interquartile range (IQR), respectively. For categorical data, absolute frequencies and percentages are presented. Associations of binary variables were calculated by use of Fisher's exact test. Overall survival (OS) and PFS estimations were plotted by Kaplan Meier method, and differences between groups were compared using log‐rank test. *P*‐values less than 0.05 were considered statistically significant (two‐sided).

## 3 RESULTS

We could identify a total of 50 patients who received LEN‐based treatment for histologically verified MALT lymphoma at our institution. Median age at treatment start was 67 years (range; 33‐85 years); 62% of patients were female and 38% male. At initial diagnosis, 32% of patients had primary gastric involvement, while the majority presented with primary extragastric MALT lymphoma (68%). The most common extragastric manifestation were the ocular adnexa (30%, 15/50), followed by MALT lymphoma of the lung (10%, 5/50) and parotid gland (8%, 4/50), and one patient each had primary of the breast, colon, skin, liver, and small intestine. Furthermore, five patients (10%) had multiple organ involvement at first diagnosis. In terms of Ann Arbor stage at LEN‐treatment start, 66% were rated as localized disease (stage IE or IIE) and 34% had disseminated disease (stage IIIE or IV). A history of autoimmune disease was present in 24% of patients. Risk stratification according to the MALT‐IPI scoring system resulted in 26% low risk, 62% intermediate risk, and 12% high risk patients. See Table 1 for more detailed patient's characteristics.

**Table 1:** Baseline characteristics overall collective (n = 50 patients)

Sex (female/male)	62% (31/50)/38% (19/50)
Median age (range)	67 years (33‐85 years)
Primary localization of MALT lymphoma	
Gastric	32% (16/50)
Extragastric	68% (34/50)
Ocular adnexa	30% (15/50)
Lung	10% (5/50)
Parotid gland	8% (4/50)
Breast	2% (1/50)
Colon	2% (1/50)
Skin	2% (1/50)
Small intestine	2% (1/50)
Liver	2% (1/50)
Multiple organ involvement	8% (5/50)
Stage of disease	
Localized disease (Ann Arbor I‐II)	66% (33/50)
Disseminated disease (Ann Arbor III‐IV)	34% (17/50)
MALT‐IPI	
Low risk	26% (13/50)
Intermedia risk	62% (31/50)
High risk	12% (6/50)
Further clinical features	
Autoimmune disorder	24% (12/50)
Plasmacellular differentiation	28% (14/50)
LDH > upper normal limit	6% (3/50)
B2M > upper normal limit	52% (26/50)
Prior treatment	48% (24/50)
Prior systemic treatment	28% (14/50)
Treatment regimen	
Lenalidomide monotherapy	32% (16/50)
R‐Lenalidomide	68% (34/50)
Median follow‐up time (interquartile range)	68.0 mo (59.3‐77.7)

Abbreviations: LDH, lactate dehydrogenase; MALT‐IPI, MALT lymphoma prognostic index; R, rituximab; UNL, upper normal limit.

The median time from initial diagnosis to treatment start with LEN‐based treatment was 5.9 months (IQR; 1.8‐36.5), and while extragastric patients were directly eligible for LEN‐treatment, patients with gastric MALT lymphoma and presence of HP had been pretreated with adequate antibiotic eradication therapy. However, we also included two patients (4%) with HP‐negative gastric MALT lymphoma as assessed by histology and serology, receiving up‐front LEN‐based therapy. Analyzed for the entire cohort, LEN was applied as first‐line treatment in 52% (26/50), while the remaining patients had been pretreated including a total of 28% (14/50) who had received prior systemic treatment for MALT lymphoma, ie, chemotherapy and/or immunotherapy.

In terms of treatment schedule and initial response rates as published previously, 32% (16/50) of patients were treated within the LEN monotherapy trial and 68% (34/50) with the combination of R‐LEN. Initial ORR for the entire cohort was 72%, with 48% achieving a CR, 24% a PR and 22% SD as best outcome, while 6% (three patients) progressed during treatment. In the entire cohort, median time to documentation of best response was 5.1 months (IQR; 2.9‐6.7), and remarkably four patients (8%) improved their outcome during prolonged follow‐up and converted to a better classification of response after the last restaging within the study protocol, including three patients with delayed CR at 12 to 32 months after LEN‐start and one converting from SD to PR after 11 months. Thus, the final outcome according to best response documented during follow‐up was 74% ORR, 54% CRs, and 20% SDs. ORR and CR rates were higher for the combination group being 77% versus 69%, and 62% versus 38%; however, none of these differences reached statistical significance (*P* = .731 and *P* = .136, respectively). There was no difference of frequency in response in terms of primary localization (ORR extragastric 74% versus gastric 75%, *P* = .912) or disease dissemination status (ORR localized 76% versus disseminated 71%, *P* = .741).

**Figure 1:**
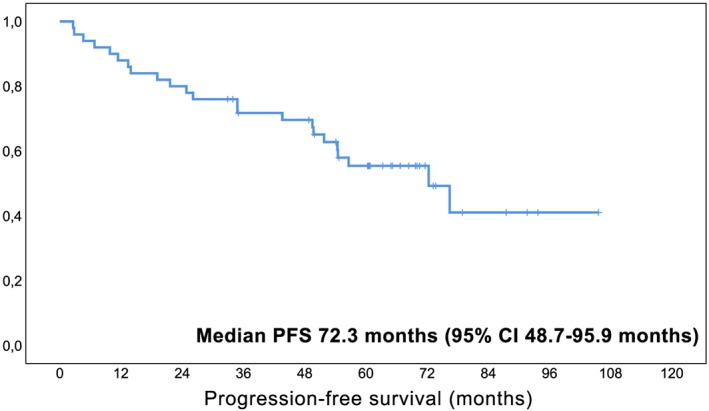
Kaplan‐Meier curve for estimated progression‐free survival in MALT lymphoma patients treated with lenalidomide‐based therapy (n = 50). *Legend. X‐axis follow‐up in months; y‐axis cumulative progression‐free survival*

**Figure 2:**
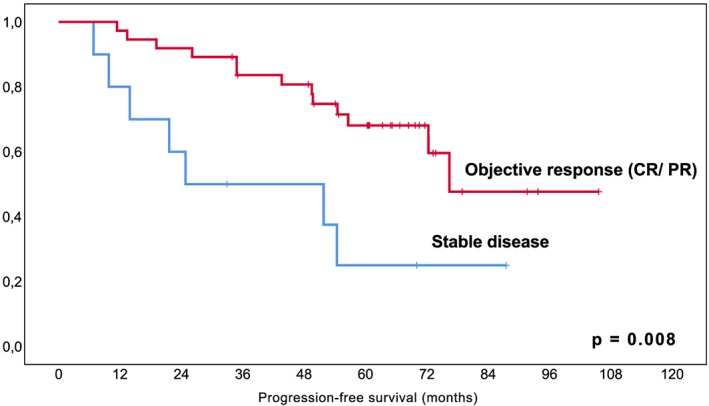
Kaplan‐Meier curve for estimated progression‐free survival in MALT lymphoma patients with objective response versus stable disease following lenalidomide. *Legend. X‐axis follow‐up in months; y‐axis cumulative progression‐free survival*

**Figure 3:**
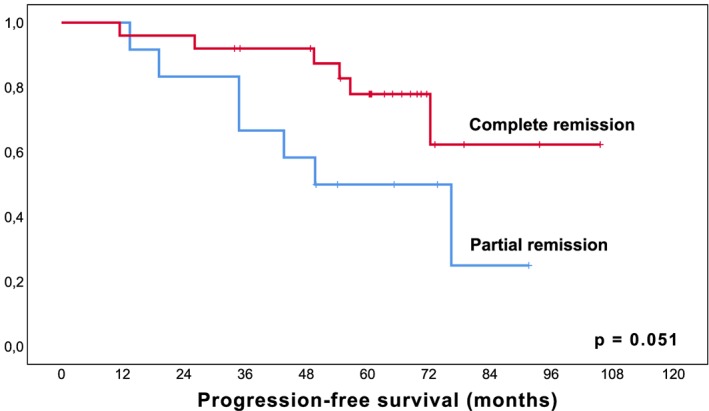
Kaplan‐Meier curve for estimated progression‐free survival in MALT lymphoma patients with complete remission versus partial remission following lenalidomide. *Legend. X‐axis follow‐up in months; y‐axis cumulative progression‐free survival*

To date, at a median follow‐up of 68 months (IQR; 59.3‐77.7 months), a total of 46% (23/50) of patients have relapsed with a median estimated PFS of 72.3 months (95%CI 48.7‐95.9 months, Figure 1) including the three patients reported as PD during the initial study observation period. Median time to relapse in patients with documented PD was 26.1 months (IQR; 11.4‐51.8). In detail, progression was local growth of residual tumor in 52% (12/23), a local relapse after CR in 30% (7/23), and a distant relapse in 17% (4/23). In the entire collective, a total of 21 patients received subsequent treatment including immuno‐/chemotherapy in 67% (14/21), local therapy in 19% (4/21), and radioimmunotherapy in 14% (3/21). The median time to next treatment was 21.5 months (IQR; 5.2‐47.5). Importantly, patients who achieved a response, i.e. CR or PR, had a significantly longer PFS than patients with SD as best response (*P* = .008, Figure 2), and patients with CR had a longer PFS than patients with PR (*P* = .051, Figure 3), suggesting an impact of depth of remission on the duration of response.

**Figure 4:**
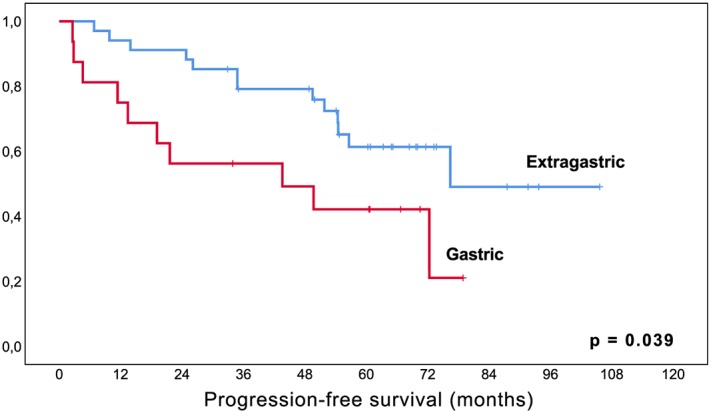
Kaplan‐Meier curve for estimated progression‐free survival in patients with primary gastric versus extragastric MALT lymphoma treated with lenalidomide Legend. X‐axis follow‐up in months; y‐axis cumulative progression‐free survival

There was no difference in PFS in terms of dissemination, i.e. localized versus disseminated disease (*P* = .668), MALT‐IPI scores (*P* = .186), or presence of an autoimmune disease (*P* = .279), but patients with primary extragastric MALT lymphoma at initial diagnosis had statistically a longer estimated PFS than patients with gastric origin (*P* = .039, Figure 4). This, however, needs to be seen within the context of the high rate of pretreated patients in the latter cohort due to previous HP‐eradication (significantly more patients pretreated in the cohort of gastric MALT lymphoma, 88% versus 29%, *P* < .001), and in view of the limited number of cases included in this series. As expected, there was a trend toward worse PFS in patients who had received prior treatment but without reaching statistical significance (*P* = .083). Prior immuno‐/chemotherapy did not significantly influence PFS (*P* = .155).

In line with the nature of the disease, OS was excellent with an estimated 5‐year OS of 92%. In terms of disease status, 50% (25/50) are currently alive without evidence of lymphoma, 38% (19/50) are alive with residual MALT lymphoma, and six patients (12%) have died including two (4%) lymphoma‐related deaths: one patient with heavily pretreated disseminated disease and one with transformation to refractory diffuse large B‐cell lymphoma (DLBCL). In addition to the later patient, another patient showed histological transformation for a total transformation rate of 4%. PCR‐based clonality analysis, however, revealed that only one of these DLBCLs was clonally related to the primary MALT lymphoma (localized gastric transformation and currently ongoing remission after R‐CHOP), while the second showed no alignment of immunoglobulin‐rearrangement pointing to a clonally unrelated, secondary manifestation of DLBCL.

## 4 DISCUSSION

Application of LEN has become a widely used approach in various lymphoid malignancies including follicular lymphoma in combination with rituximab, as well as in mantle cell lymphoma.^25^, ^27^, ^30^ In this article, we present our long‐term experience with patients suffering from MALT lymphoma undergoing a chemo‐free immunomodulatory therapy based on the application of the IMiD LEN either as monotherapy (n = 16) or in combination with rituximab (n = 34). As already seen in other trials performed at our institution, the majority of patients had extragastric MALT lymphoma, with only 32% having gastric MALT lymphoma. However, this trend is not only seen at our institution,^31^ but also in larger international studies.^32^ Whether this reflects an increase in awareness and higher diagnostic yield in extranodal MALT lymphomas or a decline in the rate of gastric MALT lymphomas is an ongoing debate. As opposed to other studies on MZL, also patients with localized disease were treated within the context of the current analysis.

Activity of LEN‐based therapy was high, with an ORR of 74% (including 54% CR rate) and 20% SD as best outcome, while 6% (three patients) progressed during treatment. Whereas ORR and particularly CR rates were higher for the combination group (77% versus 69%, and 62% versus 38%), these differences were not statistically significant. Importantly, a total of four patients showed delayed responses including three patients initially rated as PR converting to a CR at 12 to 32 months, highlighting the potential of late remissions following immunomodulatory treatment in such an indolent disease, a phenomenon that has been reported not only for LEN but also thalidomide in MALT lymphoma patients before.^33^

In terms of long‐term outcome, at a median follow‐up time of 68 months, 54% of patients are free of progression, and the estimated PFS was 72 months in our study. Interestingly, our data suggest a statistically superior PFS for patients achieving a response to LEN‐based therapy over patients with SD only (*P* = .01), and in addition PFS following CR was also longer over PR (*P* = .05). This suggests an influence of depth of remission on PFS following LEN‐based therapy, while currently there is no clear‐cut influence of response quality in patients with gastric MALT lymphoma following HP‐eradication as judged from two series in the literature.^34^, ^35^ In view of these findings, definition of prognostic parameters for response to LEN would be highly welcome. For the time being, however, there are no pathological or strict clinical criteria, and also immunohistochemical assessment of cereblon or MUM‐1 expression has not been useful with regards to this question.^36^

Finally, activity of LEN was not influenced by stage, prior immuno‐/chemotherapy and MALT‐IPI, but patients with primary gastric MALT lymphoma had a significantly shorter PFS than patients with extragastric lymphoma, which might be explained by the fact that almost all patients with gastric lymphoma had already been pretreated at least with HP‐eradication before being included into therapy with a LEN‐based regimen. Furthermore, it is not feasible to draw any strong conclusions following this observation due to the limited number of cases included in this series, precluding also direct comparison of the two different treatment regimens used. The fact that also patients with localized disease had excellent outcomes is in league with other studies performed at our institution with various systemic approaches^31^, ^37^, ^38^ and is also in line with the recommendation in guidelines that systemic therapy can also be considered in localized disease.^8^, ^9^, ^10^

The median follow‐up time (68 months) for our patient collective is comparable to a recently published analysis for R‐LEN in patients with MZL from the MD Anderson, who reported on 30 patients with median follow‐up of 75 months.^26^ In this series, however, all patients were therapy‐naive, and the majority (18/30) had nodal MZL, with only 11 cases of MALT lymphoma stage III/IV being included. Consequently, no meaningful subgroup analysis was possible, but for the whole collective also a high rate of response (93% ORR with 70% CR/CR unconfirmed) and a median PFS of 59.8 months were reported. At last follow‐up, however, the relapse rate was comparable to our cohort with 41% of patients having a documented progression/recurrence. Both our series underline the importance of long‐term follow‐up especially in indolent lymphoma entities beyond the specified study protocol, as rate of recurrence as well as time to relapse might be clinically more relevant than response rates only, which are extrapolated from phase II studies.

## CONFLICT OF INTEREST

The authors certify that they have no conflicts of interest to disclose in the subject matter discussed in this paper.

## AUTHORSHIP

Design of the study: B.K., M.R. Collection of data: B.K., W.L., O.N., M.E.M., I.S.K., M.R. Interpretation of data: B.K., W.L., O.N., M.E.M., I.S.K., M.R. Manuscript design and writing: B.K., M.R. Manuscript approval and final editing: all authors.
